# Tissue-Specific Expression Analysis and Functional Validation of *SiSCR* Genes in Foxtail Millet (*Setaria italica*) Under Hormone and Drought Stresses, and Heterologous Expression in *Arabidopsis*

**DOI:** 10.3390/plants14142151

**Published:** 2025-07-11

**Authors:** Yingying Qin, Ruifu Wang, Shuwan Chen, Qian Gao, Yiru Zhao, Shuo Chang, Mao Li, Fangfang Ma, Xuemei Ren

**Affiliations:** 1 Houji Laboratory in Shanxi Province, College of Life Sciences, Shanxi Agricultural University, Jinzhong 030801, China; 18786533470@163.com (R.W.); chensw27999@163.com (S.C.); 17503368138@163.com (S.C.); limao28ban@sxau.edu.cn (M.L.);; 2 Department of Basic Sciences, Shanxi Agricultural University, Jinzhong 030801, China; 3 College of Agriculture, Shanxi Agricultural University, Jinzhong 030801, China

**Keywords:** *Setaria italica*, SCARECROW, expression pattern, abiotic stress signaling, ABA signaling, bioinformatic analysis

## Abstract

The SCARECROW (SCR) transcription factor governs cell-type patterning in plant roots and Kranz anatomy of leaves, serving as a master regulator of root and shoot morphogenesis. Foxtail millet (*Setaria italica*), characterized by a compact genome, self-pollination, and a short growth cycle, has emerged as a C_4_ model plant. Here, we revealed two *SCR* paralogs in foxtail millet—*SiSCR1* and *SiSCR2*—which exhibit high sequence conservation with *ZmSCR1/1h* (*Zea mays*), *OsSCR1/2* (*Oryza sativa*), and *AtSCR* (*Arabidopsis thaliana*), particularly within the C-terminal GRAS domain. Both *SiSCR* genes exhibited nearly identical secondary structures and physicochemical profiles, with promoter analyses revealing five conserved *cis*-regulatory elements. Robust phylogenetic reconstruction resolved *SCR* orthologs into monocot- and dicot-specific clades, with *SiSCR* genes forming a sister branch to *SvSCR* from its progenitor species *Setaria viridis*. Spatiotemporal expression profiling demonstrated ubiquitous *SiSCR* gene transcription across developmental stages, with notable enrichment in germinated seeds, plants at the one-tip-two-leaf stage, leaf 1 (two days after heading), and roots during the seedling stage. Co-expression network analysis revealed that there is a correlation between *SiSCR* genes and other functional genes. Abscisic acid (ABA) treatment led to a significant downregulation of the expression level of *SiSCR* genes in Yugu1 roots, and the expression of the *SiSCR* genes in the roots of An04 is more sensitive to PEG6000 treatment. Drought treatment significantly upregulated *SiSCR2* expression in leaves, demonstrating its pivotal role in plant adaptation to abiotic stress. Analysis of heterologous expression under the control of the 35S promoter revealed that *SiSCR* genes were expressed in root cortical/endodermal initial cells, endodermal cells, cortical cells, and leaf stomatal complexes. Strikingly, ectopic expression of *SiSCR* genes in *Arabidopsis* led to hypersensitivity to ABA, and ABA treatment resulted in a significant reduction in the length of the meristematic zone. These data delineate the functional divergence and evolutionary conservation of *SiSCR* genes, providing critical insights into their roles in root/shoot development and abiotic stress signaling in foxtail millet.

## 1. Introduction

Foxtail millet (*Setaria italica*), a C_4_ cereal crop domesticated from its wild progenitor green foxtail (*Setaria viridis*) approximately 11,000 years ago in China, served as a cornerstone species in ancient northern Chinese agriculture and played a pivotal role in shaping Asian agricultural civilizations [1,2,3]. This diploid species has emerged as an exemplary model system for C_4_ crop research due to its inherent advantages: compact genome, short life cycle (2–3 months), high genetic diversity, self-pollination habit, and exceptional drought tolerance with remarkable water-use efficiency [2,4,5]. These agronomically valuable traits, coupled with their evolutionary significance in domestication studies, make foxtail millet both a resilient cereal crop and an ideal organism for investigating genetic mechanisms underlying plant adaptability and productivity.

Transcription factors (TFs) are regulatory proteins that govern the transcriptional level of a specific set of target genes [6]. GRAS is an important plant-specific gene family of transcription factors, named after the three known members (GAI, RGA, and SCR). In plants, the GRAS transcription factors play critical roles in numerous biological processes, such as C_4_ Kranz initiation, root radial patterning, axillary meristem initiation, gibberellin signal transduction, shoot meristem maintenance, and phytochrome A signal transduction [7,8]. As a member of the GRAS protein family, *SCARECROW* (*SCR*) genes assume crucial responsibilities and exert significant impacts in the developmental processes of both roots and shoots [9,10].

In root development, the *SCR* genes are essential for the cell radial patterning, the quiescent center (QC) maintenance, and the endodermis differentiation [11,12,13]. *AtSCR* is expressed in the cortex/endodermal initial cells and their descendant endodermal cell lineage, playing an essential role in regulating the asymmetric division of the cortex/endodermis progenitor cell in the *Arabidopsis thaliana* roots. Mutations of the *AtSCR* gene lead to the loss of a ground tissue layer, resulting in a heterogeneous cell type in the roots [10,14]. *AtSCR* is also involved in positioning and maintaining the stem cell niche in the *Arabidopsis* root meristem. It is required for the distal specification of the quiescent center (QC), which in turn regulates the fate of surrounding stem cells [12]. Additionally, *AtSCR* ensures telomere integrity, which is crucial for stem cell renewal and genome stability. Mutations in *AtSCR* lead to reduced expression of telomere-associated genes and increased DNA damage, further highlighting its role in maintaining stem cell health [15]. The function of *SCR* is conserved across different plant species, such as maize (*Zea mays*) and rice (*Oryza sativa*). The expression of *ZmSCR* was detected early during embryogenesis and was localized to the endodermal lineage in the root, indicating a gradual regionalization of expression. Recent findings in maize show that *ZmSCR* paralogs are also expressed in the cortex and stele. Furthermore, *ZmSCR* can complement the *Atscr* mutant phenotype, suggesting functional conservation [9,16]. In the rice root tip, *OsSCR* expression was observed in the endodermal cell layer and demonstrated downregulation in the daughter cortex cell following asymmetric division, in a manner analogous to that of *AtSCR* [13].

Shoot system studies unveil functional diversification of SCR proteins. *SCR* regulates the development of distinct cell types during leaf development: bundle-sheath in *Arabidopsis*, mesophyll in maize, and stomatal development early stage in rice [17]. Analysis of the shoot phenotype of *Atscr* mutants in *Arabidopsis* unveiled that both the hypocotyl and the shoot inflorescence exhibit a radial pattern defect and a loss of the normal starch sheath layer, and as a result, they are incapable of sensing gravity in the shoot [10]. *ZmSCR* is required to establish and/or maintain photosynthetic capacity in maize leaves and plays a role in the development of Kranz anatomy in maize [18,19,20]. *ZmSCR* transcripts accumulate in ground meristem cells of developing leaf primordia [19]. Mutations in the *ZmSCR* gene lead to compromised mesophyll cell development, such as proliferation of bundle sheath cells, abnormal differentiation of bundle sheath chloroplasts, vein disorientation, and many veins having sclerenchyma above and/or below instead of mesophyll cells, and so on, which affects the overall function of the leaves [18,19,20]. In rice, expression of *OsSCR* was observed in stomatal and ligule formation in leaf primordia. *OsSCR* is involved in the asymmetric division during stomata and ligule formation by establishing the polarization of cytoplasm [13]. Besides flowering plants, research on the *PpSCR1* gene of the moss *Physcomitrium patens* has revealed that its involvement is in the development of the leaf blade and mid-vein of moss [21].

Despite these advances, characterization of *SiSCR* genes in foxtail millet remains completely unexplored. To address this knowledge gap, we conducted gene structure and conserved motif analysis, physicochemical property determination, *cis*-acting element prediction in the promoter, phylogenetic tree analysis among species, and co-expression network analysis in this study. We also carried out detailed tissue-specific expression pattern analysis, in which we analyzed the expression levels of the two identified *SiSCR* genes in roots under hormone treatments (ABA, IAA, GA_3_, MeJA) and PEG6000 treatment, in leaves under drought treatment, and in panicles. Additionally, functional validation of *SiSCR* genes was conducted by cloning and heterologous expression in *Arabidopsis*. This research offers valuable insights that enable us to gain a more comprehensive understanding of the characteristics and functions of *SiSCR* genes. Moreover, it establishes a solid basis for our subsequent investigation into the roles of *SiSCR* genes in foxtail millet.

## 2. Results

### 2.1. Sequence and Characterization Analysis of the SiSCR Genes in Foxtail Millet

**Figure 1:**
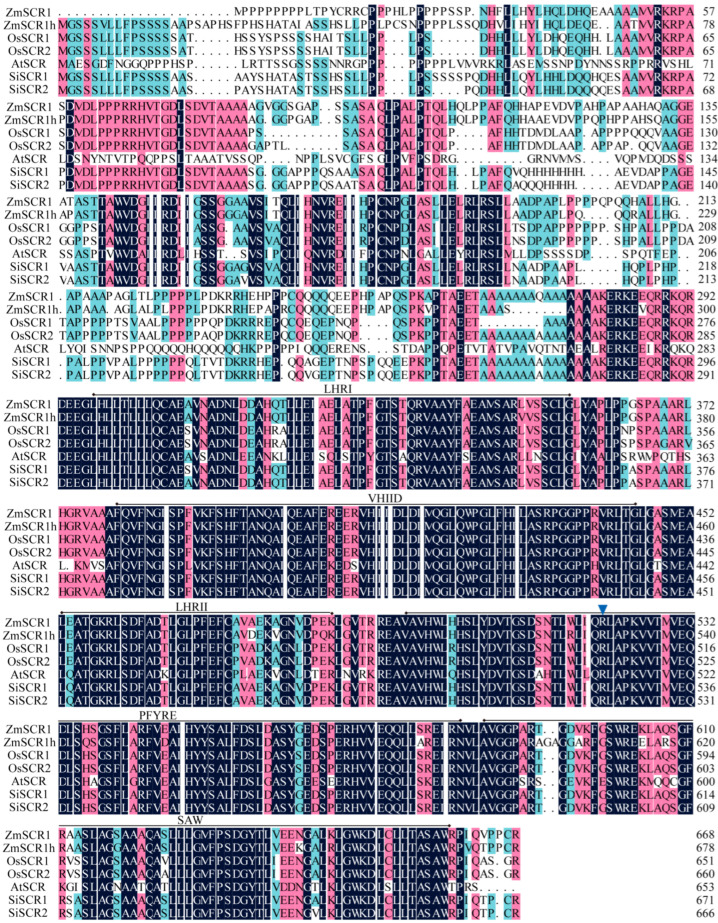
Alignment of amino acid sequences of SiSCR1/2, ZmSCR1/1h, OsSCR1/2, and AtSCR proteins. Identical amino acids are indicated by white characters in a black background. The motifs are indicated by solid bars with diamond ends. The blue arrowhead indicates that the location is conserved among these genes.

Two putative *SiSCR* genes, Si7g30800 and Si8g01880, were identified in foxtail millet by analyzing multiple foxtail millet genomic databases and the amino acid sequences of SCR proteins from *Zea mays*, *Oryza sativa*, and *Arabidopsis thaliana* [11,13,14]. According to their chromosomal localization, we named them *SiSCR1* and *SiSCR2*, respectively (Figure S1A). Both of them contain the conserved domains of the GRAS superfamily (Figure S1B). The amino acid sequence similarity between them is as high as 97.76%. As shown in Figure 1, the *SiSCR1* gene encodes a 671 amino acid putative protein, with 79.09%, 78.81%, 77.98%, 80.56% and 55.29% identity to *ZmSCR1*, *ZmSCR1h*, *OsSCR1*, *OsSCR2*, and *AtSCR*, respectively. Similarly, the *SiSCR2* gene encodes a 666 amino acid putative protein, sharing 80.00%, 78.90%, 78.51%, 80.95% and 55.26% identity with *ZmSCR1*, *ZmSCR1h*, *OsSCR1, OsSCR2,* and *AtSCR*, respectively. Although the N-terminal regions of SiSCR1/2, ZmSCR1/1h, OsSCR1/2, and AtSCR proteins are divergent, the C-terminal regions are highly conserved and include Leucine Heptad Repeat I (LHR I), VHIID motif, Leucine Heptad Repeat II (LHR II), PFYRE motif, and SAW motif as previously described [7,22]. Importantly, the order of these motifs within each protein is the same, and the positions of the exon/intron boundaries are conserved in these genes (Figure 1). This is consistent with the hypothesis that *SiSCR1* and *SiSCR2* are the orthologs of *ZmSCR1/1h*, *OsSCR1/2*, and *AtSCR*.

Then, the physicochemical properties of *SiSCR* genes were analyzed, and the results revealed that the coding sequences (CDS) of *SiSCR1* and *SiSCR2* are 2016 bp and 2001 bp, with the predicted molecular weights of 71.78 kDa and 71.12 kDa, respectively (Table 1). The SiSCR peptides were acidic, with isoelectric points of 6.06 and 5.97, respectively. The stability of both SiSCR1 and SiSCR2 proteins was identical, with the instability coefficients of 56.39 and 57.40, respectively. Based on the protein hydrophobicity index (grand average of hydropathicity, GRAVY) and the analysis of protein hydrophilicity and hydrophobicity, SiSCR1 and SiSCR2 proteins had negative hydrophilic coefficients and both of them had more hydrophilic amino acids, indicating that they are hydrophilic proteins (Table 1, Figure S2A,B). The comprehensive analysis of transmembrane domains and signal peptides revealed the absence of both transmembrane domains and signal peptides in the SiSCR peptide sequences (Figure S2C–F).

### 2.2. Gene Structure and Conserved Motif Characterization of SiSCR Genes in Foxtail Millet

**Figure 2:**
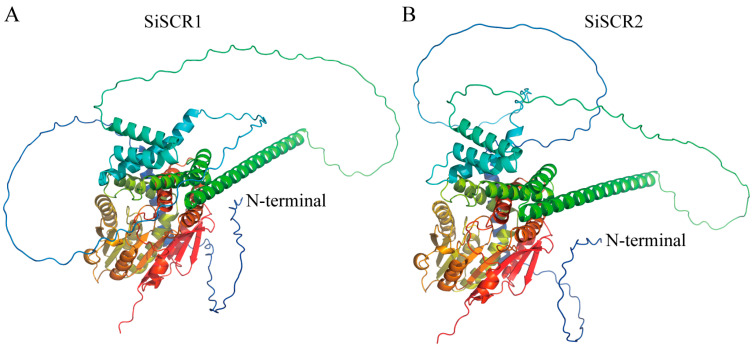
The three-dimensional structure of SiSCR proteins in foxtail millet.

A comprehensive analysis of the gene structure of the *SiSCR* genes was conducted to gain a deeper understanding of their potential functions. The analysis results showed that the gene structures of the two *SiSCR* genes were similar, with both having 3′ and 5′ non-coding regions, as well as two exons and one intron (Figure S3A). The secondary structural analysis of SiSCR proteins further revealed a notable similarity between the two proteins, with α-helices constituting 47.30% and 46.05% of their respective structures, followed by random coils at 39.79% and 41.13%, extended strands accounting for 18.91% and 9.24%, respectively, and β-sheets comprising the smallest proportion at 3.60% and 3.58%, respectively (Table 2). This composition emphasizes the relatively stable structural configuration of SiSCR proteins. The three-dimensional models of the SiSCR proteins revealed that they had complex folded structures composed of multiple secondary structural elements (Figure 2). Although both SiSCR proteins exhibited similarities in overall spatial configurations and conserved functional regions, they showed relatively significant differences in their N-terminal peptide sequences (Figure 2). In addition, the SiSCR proteins had similar structures and motif compositions. Using the MEME online platform [23], we identified 10 conserved motifs in the peptide sequence of SiSCR proteins, with lengths ranging from 21 to 50 amino acid residues (Figures S3B and S4). The locations of the 10 conserved motifs are similarly distributed, with the C-terminal conserved motifs being more compact (Figure S3B). This observation suggests that *SiSCR* may play similar functional roles in the physiological processes of plants.

### 2.3. Cis-Regulatory Elements in SiSCR Gene Promoters

**Figure 3:**
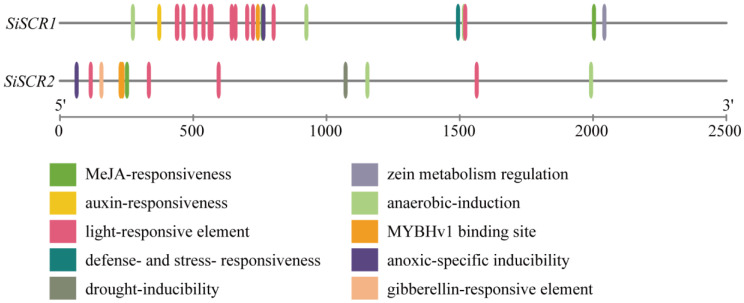
*Cis*-regulatory elements in *SiSCR* gene promoters. The elements are displayed in different colors.

To further elucidate the potential regulatory mechanisms of *SiSCR* genes in the growth, development, and stress response of foxtail millet, upstream 2500 bp sequences of *SiSCR* genes in foxtail millet were extracted for analysis using the PlantCare website [24]. A total of ten *cis*-acting elements were identified, specifically eight in the promoter region of *SiSCR1* and seven in the promoter region of *SiSCR2* (Figure 3). The ten elements included plant hormone-responsive elements such as Methyl-jasmonic acid (Me-JA), auxin, and gibberellin; environmental-responsive elements like light-responsive element, defense- and stress-responsive element, drought-inducible element, anaerobic-induction element, anoxic-specific inducible element; as well as elements involved in zein metabolism regulation and MYBHv1 binding site. Among the ten *cis*-acting elements, five elements, namely the MeJA response element, light response element, anaerobic induction element, MYB binding site, and hypoxia-specific induction element, are shared by two genes. This indicates that the expression of *SiSCR* genes is induced by oxygen content and plays a crucial role in the response of foxtail millet to MeJA and light. Moreover, the promoter of *SiSCR1* specifically contains auxin response elements, defense and stress response elements, and zein metabolism regulatory elements, which suggests that this gene is implicated in the stress resistance and metabolic regulation of foxtail millet. On the other hand, the drought-inducible element and gibberellin response element are exclusively found in the promoter of *SiSCR2*, indicating that this gene not only participates in the stress resistance response of foxtail millet but also plays a significant role in the gibberellin response process. These findings suggest that *SiSCR* genes are not only involved in hormone response pathways but also exhibit responsiveness to various environmental factors.

### 2.4. Phylogenetic Analysis of SCR Proteins in 16 Plant Species

**Figure 4:**
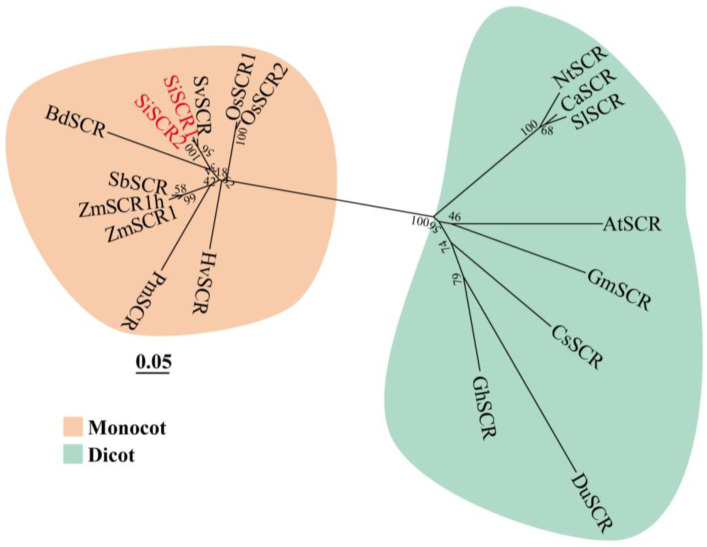
Neighbor-joining phylogenetic tree of SCR proteins from 16 plant species. Abbreviations for each species and accession numbers for genes are as follows: *SiSCR1/2*: *Setaria italica* (Si7G30800/Si8G01880); *SvSCR*: *Setaria viridis* (XP_034604466.1); *PmSCR*: *Panicum miliaceum* (RLM58022.1); *ZmSCR1/1h*: *Zea mays* (NP_001168484.1/NP_001336868.1); *SbSCR*: *Sorghum bicolor* (XP_021317511.1); *OsSCR1/2*: *Oryza sativa* (LOC_Os11g03110.1/LOC_Os12g02870.1); *HvSCR*: *Hordeum vulgare* (XP_044948410.1); *BdSCR*: *Brachypodium distachyon* (XP_010239549.2); *CaSCR*: *Capsicum annuum* (XP_016545671.2); *SlSCR*: *Solanum lycopersicum* (XP_010327696.1); *NtSCR*: *Nicotiana tabacum* (XP_016486111.1); *AtSCR*: *Arabidopsis thaliana* (AT3G54220); *GmSCR*: *Glycine max* (KAH1235198.1); *CsSCR*: *Cucumis sativus* (NP_001295787.1); *DuSCR*: *Dalzellia ubonensis* (LAD56344.1); *GhSCR*: *Gossypium hirsutum* (XP_016715277.2). The phylogenetic tree was constructed with the neighbor-joining method using MEGA7.

To explore the evolutionary relationship between SiSCR proteins in foxtail millet and SCR proteins in other species, the amino acid sequences of SCR proteins from 15 other plant species were retrieved from the NCBI database, including *Setaria viridis*, *Panicum miliaceum*, *Zea mays*, *Sorghum bicolor*, *Oryza sativa*, *Hordeum vulgare*, *Brachypodium distachyon*, *Capsicum annuum*, *Solanum lycopersicum*, *Nicotiana tabacum*, *Arabidopsis thaliana*, *Glycine max*, *Cucumis sativus*, *Dalzellia ubonensis*, and *Gossypium hirsutum*. We aligned the amino acid sequence of the SiSCR protein in foxtail millet with homologous sequences in other species and constructed a phylogenetic tree using the neighbor-joining method (Figure 4 and Table S1). The phylogenetic tree primarily consists of two branches: one branch clusters monocot SCR proteins, while the other branch clusters dicot SCR proteins. The amino acid sequence of foxtail millet SiSCR proteins is highly related to gramineous plants. Particularly, it has the most intimate relationship with SvSCR protein of its ancestral species, *Setaria viridis*.

### 2.5. Expression Pattern Analysis of SiSCR Genes in Foxtail Millet

**Figure 5:**
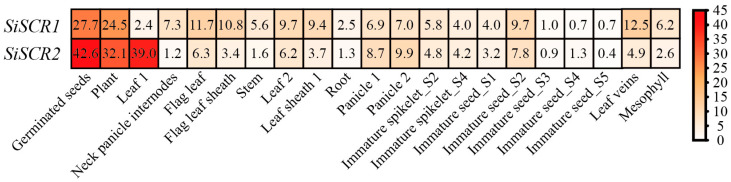
Expression pattern analysis of *SiSCR* genes in different tissues. The heatmap of their expression profiles across developmental stages was generated using normalized RNA-seq data from the MDSi database, with TBtools used for visualization. Color gradients from red to white indicate expression levels from high to low.

In order to comprehensively understand the function of *SiSCR* genes in foxtail millet, we analyzed the transcript level of *SiSCR* genes in different tissues (root, stem, leaf, panicle and spikelet) of Jingu 21 variety at different growth stages (3 days after germination, one tip and two leaves stage, heading stage, filling stage and panicle differentiation stage). After obtaining the transcriptome data from the MDSi database [25], we generated a heat map to display the expression pattern of *SiSCR* genes (Figure 5). The TPM values of *SiSCR* genes are listed in Table S2. The *SiSCR* genes are expressed throughout the foxtail millet’s entire growth and development process. Specifically, the *SiSCR1* gene is predominantly expressed during two stages: germinated seeds and plants at the one-tip-two-leaf stage. Its expression is also detectable in leaf veins, flag leaf, and flag leaf sheath during the filling stage. The *SiSCR2* gene is primarily expressed during three stages: germinated seeds, plants at the one-tip-two-leaf stage, and leaf 1 (two days after heading). The expression levels of *SiSCR* genes, particularly the *SiSCR2* gene, are notably low in the stem and root during the heading stage. The *SiSCR* genes are expressed in the panicle, primarily in primary panicle branches and third panicle branches at the panicle-branch differentiation stage, and also in the S2 and S4 stages of immature spikelets. In the immature seeds stage, the *SiSCR* genes exhibit the highest expression only in the S2 stage. As the seeds mature, the expression level of *SiSCR* rapidly decreases. In mature seeds, the *SiSCR* genes are no longer expressed (Figure 5 and Table S2).

In addition, we also used RT-qPCR to assess the expression patterns of *SiSCR* genes in the roots, stems, and leaves of seedlings grown for 28 days, 3-day germinated seeds, immature seeds (S2) at the middle grain filling stage, and young panicles at the early growth stage (Figure S5). The expression patterns of *SiSCR* genes in seeds germinated for 3 days, immature seeds (S2), young panicles, and stems aligned with the transcriptome data. The expression levels of *SiSCR* genes in roots during the seedling stage were significantly higher than those in roots during the grain filling stage. The expression levels of *SiSCR* genes in leaves during the seedling stage matched those in Leaf2 (Figure 5 and Figure S5).

**Figure 6:**
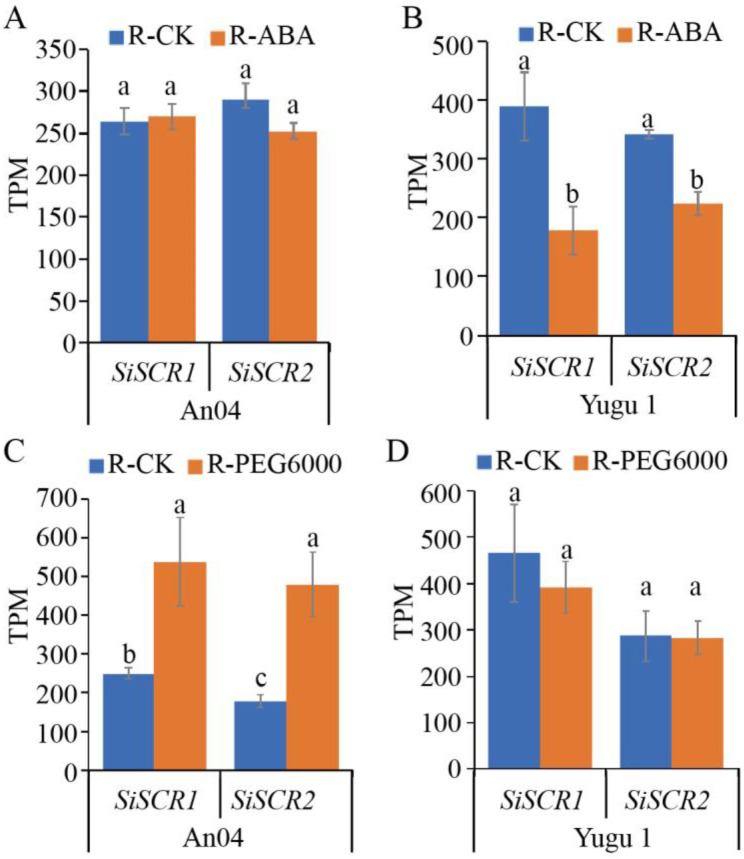
Expression patterns of *SiSCR* genes in the roots of foxtail millet. (**A**,**B**) Expression patterns of *SiSCR* genes in 9-day-old seedling roots treated with 2 µM ABA (R-ABA) and without ABA (R-CK) in An04 and Yugu1. (**C**,**D**) Expression patterns of *SiSCR* genes in 9-day-old seedling roots treated with 20% PEG6000 (R-PEG6000) and without PEG6000 (R-CK) in An04 and Yugu1. Bar graphs represent differences in transcriptome sequencing duplicates of root samples in An04 and Yugu1. Statistical significance was determined by *t*-test (lowercase letters indicate *p* < 0.05). TPM: transcripts per million.

**Figure 7:**
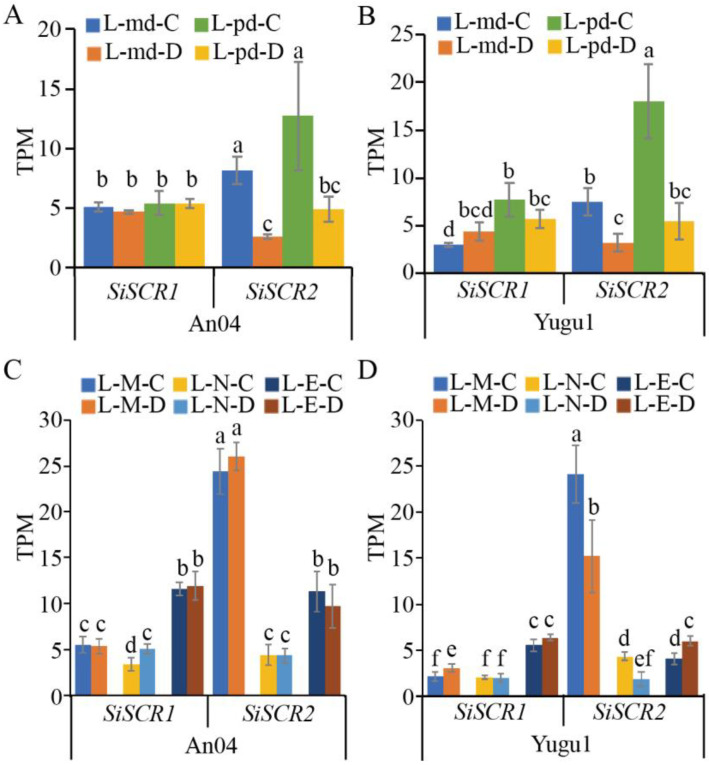
Expression patterns of *SiSCR* genes in the leaves of foxtail millet. (**A**,**B**) Expression patterns of *SiSCR* genes in the leaves (L) of 30-day-old plants, with the treatment of drought (**D**) and the untreated control (**C**) at the midday (md) and pre-dawn (pd) within an artificial climate chamber in An04 and Yugu1. (**C**,**D**) Expression patterns of *SiSCR* genes in the leaves (L) of 30-day-old plants, with the treatment of drought (D) and the untreated control (C) in the morning (M), at noon (N), and in the evening (E) of An04 and Yugu1 in the experimental field. Bar graphs represent differences in transcriptome sequencing replicate samples of leaf samples in An04 and Yugu1. Statistical significance was determined by *t*-test (lowercase letters indicate *p* < 0.05). TPM: transcripts per million.

*SCR* regulates the patterning of cell types in both the roots and Kranz leaves of plants, functioning in the endodermis immediately adjacent to the root vasculature and in the mesophyll at a two-cell distance from leaf veins [9,12,19,26]. Transcriptome sequencing was performed to investigate the expression levels of *SiSCR* genes in the roots of seedlings and leaves of plants (Figure 6 and Figure 7). ABA signaling mediates many types of abiotic stresses, such as drought, salinity, heat, and cold stress. In roots, ABA plays an essential role in inhibiting cell division and elongation, suppressing root hair growth, and root meristem activity [27,28,29]. To investigate the response of *SiSCR* genes following ABA treatment, transcriptome sequencing analysis was performed on the roots of foxtail millet seedlings subjected to ABA treatment. An04 and Yugu1 are two *Setaria italica* cultivars, with ‘An04’ defined as a drought-sensitive variety and ‘Yugu1’ defined as a drought-tolerant variety, exhibiting contrasting drought tolerance levels [30]. Our analysis revealed that the expression levels of the *SiSCR* genes in the roots of two foxtail millet cultivars were basically the same in the absence of ABA treatment. However, after ABA treatment, significant differences in the expression levels of the *SiSCR* genes were observed between the two cultivars. ABA treatment did not have a significant impact on the expression level of the *SiSCR* genes in An04 roots. In contrast, ABA treatment led to a significant downregulation of the expression level of the *SiSCR* genes in Yugu1 roots (Figure 6A,B), indicating that the response of the *SiSCR* genes to ABA treatment may vary among different foxtail millet ecotypes in roots.

Analysis of *cis*-acting elements in the promoter suggests that *SiSCR* gene expression may be regulated by auxin, gibberellin, and MeJA. Therefore, we also assessed changes in *SiSCR* gene expression levels under treatments with IAA, GA_3_, and MeJA (Figure S6). In Yugu1, IAA treatment induced no significant change in *SiSCR1* expression at 12 h, but resulted in significant decreases at 24 h and 48 h. GA_3_ treatment consistently reduced *SiSCR1* expression across all time points, with no significant difference in the magnitude of reduction. MeJA treatment also decreased *SiSCR1* expression, which was most pronounced at 24 h and 48 h. For *SiSCR2* in Yugu1, IAA treatment caused the expression level of the *SiSCR2* gene to drop sharply at 24 h and 48 h. GA_3_ treatment significantly upregulated *SiSCR2* expression at 12 h and 24 h, while at 48 h, its expression level was slightly lower than the unstressed control. MeJA treatment decreased *SiSCR2* expression at 12 h, but expression recovered to pre-treatment levels at 24 h and 48 h (Figure S6). In An04, IAA treatment significantly decreased *SiSCR1* expression only at 48 h. GA_3_ treatment had no significant effect on *SiSCR1* expression. Prolonged MeJA treatment time led to a gradual decrease in *SiSCR1* expression. For *SiSCR2* in An04, IAA treatment reduced expression at 12 h and 48 h. GA_3_ treatment increased *SiSCR2* expression at 12 h and 48 h but decreased it at 24 h. MeJA treatment increased *SiSCR2* expression at 12 h and 24 h, followed by a gradual return to pre-treatment levels at 48 h (Figure S6).

Drought stress increases the root-shoot ratio and causes plants to form more capillary roots to better absorb water [31,32]. In this regard, a hydroponic experiment was conducted on the roots of two *Setaria italica* cultivars with polyethylene glycol (PEG6000) induced water stress, and a soil drought treatment was implemented on the leaves (Figure 6C,D and Figure 7). Our results indicate that PEG6000 treatment can significantly upregulate the expression level of the *SiSCR* genes in the roots of An04 by approximately 2-fold, but has no significant effect on the expression level of the *SiSCR* genes in the roots of Yugu1 (Figure 6C,D). This suggests that the expression of the *SiSCR* genes in the roots of An04 is more sensitive to PEG6000 treatment.

SCARECROW plays a role in the development of Kranz anatomy in maize [18,19], so we conducted a transcriptomic analysis on the expression of *SiSCR* genes in the leaves of foxtail millet, given that it is also a C_4_ monocot. We, respectively, analyzed the expression levels of the *SiSCR* genes in the leaves of An04 and Yugu1, which were planted in the artificial climate chamber and the experimental field, with or without drought treatment at different time points during a day (Figure 7). The expression levels of the *SiSCR1* gene in the leaves of the two varieties showed no significant differences between the drought treatment and the untreated control under the growth conditions in the artificial climate chamber. However, the expression level of the *SiSCR2* gene decreased significantly after drought treatment, regardless of whether it was at midday or in the pre-dawn (Figure 7A,B). In addition, the expression levels of the *SiSCR* genes were significantly higher in the pre-dawn period than those at midday in Yugu1 (Figure 7A,B). Under the growth conditions in the field, the expression levels of the *SiSCR* genes in the leaves of An04 showed no significant differences between the drought treatment and the untreated control, with the exception that the expression of *SiSCR1* increased significantly after drought treatment at noon. Regarding Yugu1, under the same growth conditions in the field, the expression of *SiSCR1* in its leaves increased significantly after drought treatment in the morning, while remaining relatively stable at other time points. Meanwhile, the expression level of *SiSCR2* in the leaves of Yugu1 decreased significantly after drought treatment in the morning and at noon, but increased after drought treatment in the evening (Figure 7C,D). In addition, the expression level of the *SiSCR1* gene is the highest in the evening period, followed by that in the morning and at noon; the expression level of the *SiSCR2* gene is the highest in the morning period, followed by that in the evening and at noon (Figure 7C,D). These results indicate that the *SiSCR2* gene may be involved in the stress response induced by drought in leaves, and the expression of the *SiSCR* genes in the leaves of foxtail millet may be regulated by the circadian clock.

In addition, we also analyzed the transcriptome sequencing results of foxtail millet panicle development. There were no significant differences in the expression levels of *SiSCR* genes between the two developmental stages (stage 1 and stage 2) of young panicles (Figure S7).

### 2.6. Co-Expression Network Profiling of the SiSCR Genes in Foxtail Millet

**Figure 8:**
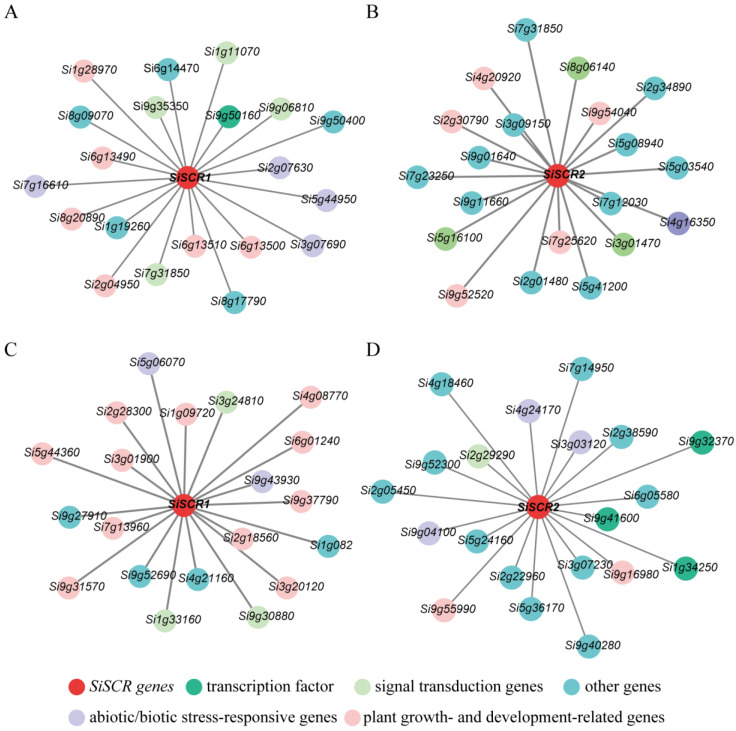
Co-expression networks of *SiSCR1* and *SiSCR2* under ABA and drought stress in Yugu1. (**A**) Network of *SiSCR1* under ABA treatment. (**B**) Network of *SiSCR2* under ABA treatment. (**C**) Network of *SiSCR1* under PEG6000-induced drought stress. (**D**) Network of *SiSCR2* under PEG6000 treatment. Nodes are color-coded by functional categories: red, core *SiSCR* genes; dark green, transcription factors; light green, signaling components; purple, abiotic stress-responsive genes; pink, growth-related genes; gray, unannotated genes. Edges represent co-expression correlations.

To elucidate the functional roles of *SiSCR* genes, we constructed a co-expression network associated with *SiSCR1/2* using the differentially expressed gene (DEG) network derived from transcriptomic data of Yugu1 seedling root tips as mentioned above. Functional annotation of the top 20 most connected DEGs in the *SiSCR1/2* co-expression networks revealed significant enrichment of plant growth/development-related, abiotic/biotic stress-responsive, and signal transduction genes, with shared compositional features under ABA/PEG6000 treatments (Figure 8). Specifically, the *SiSCR1* network under ABA contained 6 growth and development-related genes, 4 stress-related genes, and 4 signal transduction-related genes (Figure 8A), while the *SiSCR1* network under PEG6000 comprised 11, 2, and 3 genes in these respective categories (Figure 8C). Similarly, the *SiSCR2* network under ABA included 5 growth and development-related genes, 1 stress-related gene, and 3 signal transduction-related genes (Figure 8B), whereas the *SiSCR2* network under PEG6000 involved 2, 3, and 1 genes in these classes (Figure 8D). These findings indicate that there is a connection between *SiSCR* genes and other functional genes.

### 2.7. Analysis of Expression Patterns of pSuper:SiSCR1/2-GFP in Arabidopsis thaliana

**Figure 9:**
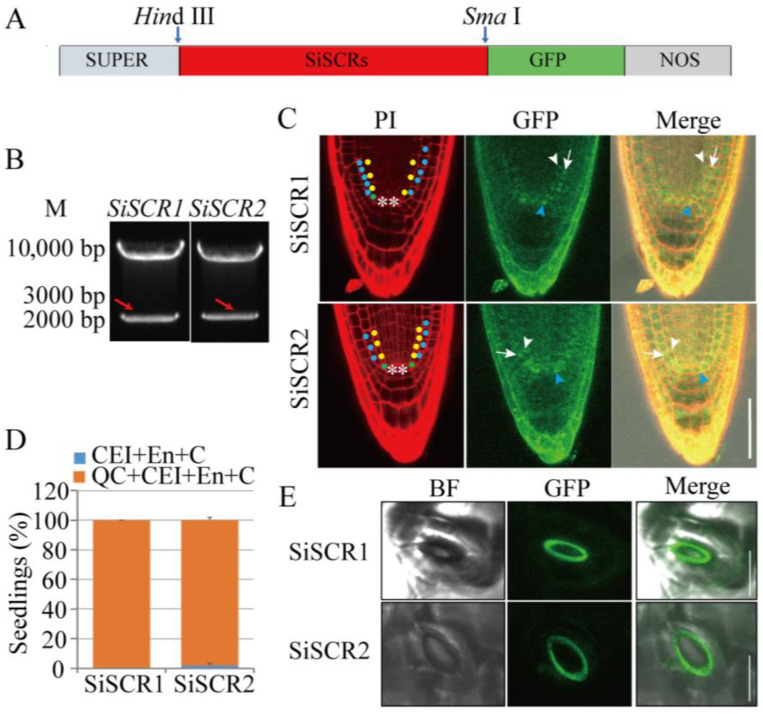
Heterologous expression analysis of *SiSCR* genes in transgenic *Arabidopsis thaliana*. (**A**) Vector construction of *pSuper:SiSCR1/2-GFP*. (**B**) Enzyme digestion identification of *pSuper:SiSCR1/2-GFP* vector. (**C**) Heterologous expression pattern of *SiSCR* genes in root tips of transgenic *Arabidopsis* (yellow dot: endodermis; blue dot: cortex; ** quiescent centre; white arrowhead: *SiSCR* genes expression in the endodermis; blue arrowhead: *SiSCR* genes expression in the cortex/endodermis initial cell; white arrow: *SiSCR* genes expression in the cortex). (**D**) Heterologous expression analysis of *SiSCR* genes in transgenic *Arabidopsis* root tips (CEI: cortex/endodermis initial cell; En: endodermis; C: cortex; QC: quiescent centre). (**E**) *SiSCR* genes were expressed in the stomata of *Arabidopsis thaliana* (BF: Bright field. Bar = 100 μm).

To gain a further understanding of the *SiSCR* genes, we analyzed their expression patterns in *Arabidopsis thaliana*. We successfully expressed the *pSuper:SiSCR1/2-GFP* construct in *Arabidopsis thaliana* and analyzed the expression pattern of the *SiSCR* genes in the T3 homozygous transgenic positive lines (Figure 9). The results showed that the expression patterns of *SiSCR1* and *SiSCR2* in *Arabidopsis thaliana* were similar. The SiSCR1-GFP and SiSCR2-GFP proteins were detected in the root cap, quiescent center cells, cortex/endodermis initial cells, endodermis, and cortex in *Arabidopsis thaliana* (Figure 9C,D). The results were similar to the expression pattern of *AtSCR* in *Arabidopsis thaliana*, which was mainly expressed in the root quiescent center cells, cortex/endodermis initial cells, and endodermis. However, the heterologously expressed *SiSCR* genes were also expressed in the root cap and cortex of *Arabidopsis thaliana*, which was different from the expression of *AtSCR* in *Arabidopsis thaliana*. In the leaves of *Arabidopsis thaliana*, *SiSCR genes* were specifically expressed in the stomata (Figure 9E).

### 2.8. Root Growth Characterization in pSuper:SiSCR1/2-GFP Transgenic Arabidopsis thaliana

**Figure 10:**
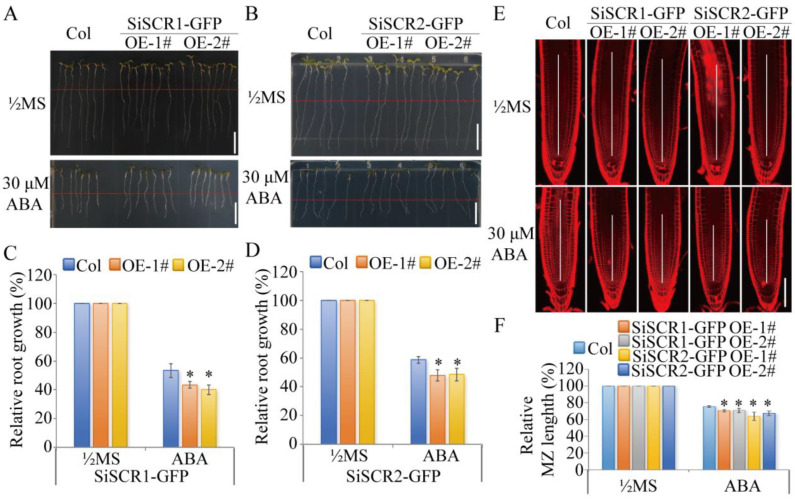
Overexpression of *SiSCR* genes reduces ABA resistance in *Arabidopsis thaliana*. (**A**) Primary root phenotypes of wild-type Col, *SiSCR1*-overexpressing lines (OE-1# and OE-2#). (**B**) Primary root phenotypes of wild-type Col, *SiSCR2*-overexpressing lines (OE-1# and OE-2#). For (**A**,**B**), seeds were germinated on ½MS medium for 4 days and then transferred to fresh ½MS medium with or without 30 μM ABA (Bar = 1 cm). (**C**) Relative primary root growth of plants in (**A**), normalized to root length on ½MS medium (set as 100%). Data represent mean ± SE (n = 3 replicates; 7 seedlings per replicate). (**D**) Relative primary root growth of plants in (**B**), normalized as in (**C**). (**E**) The root meristem zones of wild-type Col, *SiSCR1*-overexpressing lines (OE-1# and OE-2#), and *SiSCR2*-overexpressing lines (OE-1# and OE-2#) on the ½MS medium and the ½ MS medium containing 30 μM ABA (Bar = 100 μm). (**F**) Relative meristematic zone (MZ) length of plants in (**E**). All experiments were repeated three times with consistent results. Statistical significance was determined by *t*-test (* *p* < 0.05).

To elucidate the molecular functions of *SiSCR* genes, root growth assays were performed using *Arabidopsis* transgenic lines overexpressing *pSuper:SiSCR1-GFP* or *pSuper:SiSCR2-GFP* constructs. Quantitative analysis revealed that both *SiSCR1*-OE (lines OE-1# and OE-2#) and *SiSCR2*-OE (lines OE-1# and OE-2#) displayed significantly enhanced ABA-mediated root growth inhibition compared to wild-type (Figure 10A–D). The results demonstrate that ectopic expression of either *SiSCR1* or *SiSCR2* substantially amplifies ABA responsiveness in *Arabidopsis thaliana*.

Root architecture analysis of *pSuper:SiSCR1/2-GFP* transgenic plants demonstrated that exogenous ABA treatment induced a marked reduction in meristematic zone length, with both *pSuper:SiSCR1-GFP* and *pSuper:SiSCR2-GFP* transgenic lines exhibiting a pronounced shortening of root apical meristem longitudinal dimensions compared to wild-type Col (Figure 10E,F).

We also examined root phenotypes and root architectures of *SiSCR1*-OE (lines OE-1# and OE-2#) and *SiSCR2*-OE (lines OE-1# and OE-2#) under treatments with IAA, GA_3_, and MeJA (Figure S8). Quantitative analysis revealed that under IAA treatment, both root elongation and root meristem length in *SiSCR1*-OE and *SiSCR2*-OE were significantly reduced compared to the control, with the degree of reduction similar to that of wild-type Col. Under GA_3_ treatment, both *SiSCR1*-OE and *SiSCR2*-OE root systems exhibited significant elongation compared to the control but the extent of elongation was less than that of Col. The meristem zone length also significantly increased. Except for the *SiSCR2*-OE-1# line, the extent of meristem elongation in the other lines was comparable to that of Col. Under MeJA treatment, root elongation was significantly inhibited. The degree of inhibition in *SiSCR1*-OE (lines OE-1# and OE-2#) was similar to wild-type Col, whereas *SiSCR2*-OE (lines OE-1# and OE-2#) exhibited more severe inhibition than wild-type Col. For meristem growth, the meristem length in Col and *SiSCR1*-OE (lines OE-1# and OE-2#) showed no significant change, while that in *SiSCR2*-OE (lines OE-1# and OE-2#) was significantly reduced compared to the control (Figure S8).

## 3. Discussion

Foxtail millet (*Setaria italica*) is a minor but economically important crop species and an emerging model plant for C_4_ grasses [2,33]. In plants, asymmetric cell divisions can lead to distinct cell fates, giving rise to daughter cells of different sizes and thus increasing the cellular diversity within an organ. *SCR* is a transcription factor containing the GRAS domain, which controls the asymmetric periclinal cell divisions in flowering plants by regulating the radial patterning of ground tissue in roots and the cell proliferation in leaves. The *SCR* genes have been the subject of in-depth investigations in various plants, including flowering plants *Arabidopsis thaliana*, *Zea mays*, *Oryza sativa*, and moss *Physcomitrium patens* [13,17,19,20,21]. Nevertheless, research on *SCR* in foxtail millet remains severely deficient. In the current study, by means of sequence homology alignment of AtSCR, ZmSCR1/1h, and OsSCR1/2 proteins, along with the prediction of conserved domains and the analysis of conserved sequences, two *SiSCR* genes were successfully identified. The number of *SiSCR* genes in foxtail millet is consistent with that in maize and rice. The physicochemical properties and structures of the two *SiSCR* genes exhibit a high degree of similarity. Notably, both genes possess the GRAS domain as well as five conserved motifs at the C-terminal, namely Leucine Heptad Repeat I, VHIID motif, Leucine Heptad Repeat II, PFYRE motif, and SAW motif (Figure 1 and Figure S1), which are consistent with previous studies [8,21]. Moreover, the secondary structure compositions and tertiary structure conformations of SiSCR proteins manifest a remarkably striking similarity (Table 2 and Figure 2), which implies a potential functional redundancy or overlap between the two *SiSCR* genes in the modulation of plant growth, development, as well as their adaptive responses to environmental stimuli.

*SCR* plays multiple essential roles in plant growth and development. Firstly, *SCR* directly controls genes that are involved in both development and stress responses [34]. In the meristematic transition zone (TZ) of *Arabidopsis thaliana*, *AtSCR* integrates the activities of auxin, gibberellin, and cytokinin to promote root meristem development [35]. BIG, which is a regulator of polar auxin transport, is crucial for regulating the growth and development of *Arabidopsis* and mainly maintains root meristem activity and stem cell niche integrity through the *SCR/SHR* (*SHORTROOT*) pathway [36]. *SCR* and *SHR* negatively control the expression levels of three receptor-like kinases (ARH1, FEI1, and FEI2) that regulate GA biosynthesis to govern ground tissue patterning [37]. *SCR* is also involved in the process that jasmonate (JA) regulates the activity of organizer cells in the root stem cell niche [38]. Regarding the coordination between stress responses and development, it is known that stress responses in plants are tightly coordinated with developmental processes. SCR protein shows binding to regulatory regions of stress-responsive genes and regulates a set of stress response genes [39]. *Atscr* mutant was found to be hypersensitive to abscisic acid (ABA). *AtSCR* repressed *ABI4* (*ABA-INSENSITIVE 4*) and *ABI5* directly and specifically in the apical meristem [40]. Additionally, *AtSCR* plays a role in redox homeostasis and oxidative stress response in the root [41]. *SCR gene* also maintains root stem cells by promoting the expression of genes that ensure genome integrity and establishes a connection between genome integrity and stem cell maintenance in the roots of *Arabidopsis* [15]. In maize, *ZmSCR* is required to establish and/or maintain photosynthetic capacity in maize leaves [20]. The analysis of the promoter region of the *SiSCR* genes revealed ten *cis*-acting elements, including those related to abiotic stress, plant hormone responses, and plant growth and development (Figure 3). Co-expression network analysis of *SiSCR* genes also revealed significant enrichment of genes functionally linked to growth regulation and stress adaptation (Figure 8). Overexpression of *SiSCR* genes in *Arabidopsis* resulted in hypersensitivity to ABA and GA_3_, inducing shortening of the root meristem in response to ABA and elongation in response to GA_3_ (Figure 10 and Figure S8). These findings underscore the pivotal role of *SiSCR* genes in regulating plant growth and development as well as mediating stress responses.

The results of the tissue expression pattern analysis heatmap and RT-qPCR analysis indicate that the *SiSCR* genes are expressed throughout the growth and development process of foxtail millet except in matured seeds, especially in germinated seeds, plants at the one-tip-two-leaf stage, leaf 1 (two days after heading), leaf veins and roots during the seedling stage, etc. (Figure 5 and Figure S5, Table S2), which highlights the importance of the *SiSCR* genes in the process of plant growth and development. *AtSCR* modulates the growth of the apical meristem in response to ABA signals [34]. In the current study, upon ABA treatment, a remarkable reduction in the expression levels of the *SiSCR* genes was observed in Yugu1, implying that the *SiSCR* genes may be implicated in the root growth and development regulated by ABA. Nevertheless, following the ABA treatment, no significant alteration in the expression levels of the *SiSCR* genes was detected in An04 (Figure 6A,B). This finding indicates that in the two materials, Yugu1 and An04, which display disparate drought tolerance levels, the response modalities of *SiSCR* genes to ABA vary. Similarly, the *SiSCR* genes also exhibit distinct response patterns to PEG6000 in Yugu1 and An04. The expression of the *SiSCR* genes in the roots of An04 is more sensitive to PEG6000 treatment than that in Yugu1 (Figure 6C,D). The *SCR* genes also play a very important role in aspects such as the development of plant leaves and the response of photosynthesis [18,19,20]. Therefore, we analyzed the expression of the *SiSCR* genes in the leaves of Yugu1 and An04 under drought treatment conditions. In Yugu1, the expression levels of the *SiSCR* genes were significantly higher in the pre-dawn period than those at midday. Among them, the expression level of the *SiSCR2* gene decreased significantly after drought treatment, regardless of whether it was at midday or in the pre-dawn period. However, the expression level of the *SiSCR1* gene showed no significant difference between the drought treatment and the untreated control (Figure 7A,B). In the morning and at noon, the expression levels of the *SiSCR2* gene, respectively, were significantly higher than those of the *SiSCR1* gene. In the evening, the expression level of the *SiSCR2* gene either showed no significant difference from that of the *SiSCR1* gene (in An04) or was slightly lower than that of the *SiSCR1* gene (in Yugu1). In both cultivars, the expression level of the *SiSCR1* gene was the highest in the evening, followed by that in the morning and at noon, while *SiSCR2* had the highest expression in the morning, then in the evening and at noon. After drought induction, in Yugu1, the expression level of the *SiSCR2* gene was significantly downregulated in the morning and at noon and increased in the evening. However, in An04, there was no significant change in the expression level of the *SiSCR2* gene before and after drought treatment. The *SiSCR1* gene showed an increase in expression level after drought treatment at noon in An04 and an increase in expression level after drought treatment in the morning in Yugu1 (Figure 7C,D). This further illustrates that the signal networks involved in the regulation of the *SiSCR* genes in the two materials, Yugu 1 and An04, may be different.

There are functional differences in the *SCR* gene between dicotyledons and monocotyledons [17]. The division of SCR proteins from monocotyledonous and dicotyledonous plants into different subgroups on the phylogenetic tree suggests that SCR proteins most likely diverged in different groups during the early stage of angiosperm evolution (Figure 4).

In *Arabidopsis*, tomato, cucumber, and rice, *SCR* expression patterns are restricted to the endodermal cell lineage and quiescent center [14,42,43,44]. In maize, the *ZmSCR* gene is expressed in the root cortex, endodermis, and stele [16]. In legume species, *SCR* orthologs are expressed not only in the endodermis but also in the cortex and to a lesser extent in the epidermis [45]. In this study, cloned *SiSCR* genes from foxtail millet were heterologously expressed in *Arabidopsis* under the control of the 35S promoter, and their expression patterns were characterized. *SiSCR* genes were predominantly expressed in *Arabidopsis* root cortex/endodermal initial cells, endodermis, cortex, and root cap (Figure 9C,D), which are not completely consistent with the expression pattern of the *AtSCR* gene. This discrepancy may arise from the heterologous expression of the foxtail millet *SiSCR* genes, as the *SiSCR* genes exhibit relatively low homology to *AtSCR* (55.29% and 55.26%, Figure 1). Another possible reason is the employment of the 35S promoter in place of native promoters. The use of the 35S promoter might lead to altered expression patterns compared to native conditions, potentially influencing the interpretation of tissue-specificity. However, the distinct subcellular localization and partial tissue-specific expression still provide valuable insights into *SiSCR* function. Genes driven by the 35S promoter can also exhibit specific expression in defined tissues or subcellular compartments. The transgenic *Arabidopsis* plants harboring *p35S:FNRctp-GFP*, functioning as a chloroplast-localized GFP reporter line, exhibit specific expression within the chloroplast stroma of leaf cells [46]. In both transiently and stably transformed *Arabidopsis* root cells and guard cells expressing *pSuper:GHR1-GFP* (Guard cell hydrogen peroxide-resistant1), GHR1-GFP predominantly localizes to the cell surface, whereas GFP alone is primarily detected in the cytoplasm and nucleus [47]. Regarding the transgenic *Arabidopsis* plants expressing *p35S:GGPSs-GFP* (Geranylgeranyl diphosphate synthase), GGPS1/3-GFP localizes to the chloroplasts; GGPS2/4-GFP localizes to the endoplasmic reticulum; GGPS5-GFP localizes to the mitochondria [48]. Notably, while the 35S promoter can direct specific expression, its constitutive activity may not fully recapitulate the native regulatory context. In subsequent studies, we will further characterize the expression patterns of these genes in *Arabidopsis* and foxtail millet using their native promoters. In rice leaves, *OsSCR* genes have been co-opted to regulate asymmetric cell division (ACD), and this asymmetric cell division promotes the formation of guard mother cells within stomatal cell files. However, this is not the case in the leaves of *Arabidopsis* or maize [17,49]. Our results showed that *SiSCR* genes were expressed in the stomata of *Arabidopsis* leaves (Figure 9E), suggesting that the *SiSCR* genes might be involved in the development of stomata in leaves.

## 4. Materials and Methods

### 4.1. Sequence Analysis of the SiSCR Genes in Foxtail Millet

With the amino acid sequences of SCR from maize [19], rice [17], and *Arabidopsis* [14] as references, we utilized multiple foxtail millet genomic datasets to identify *Si7g30800* and *Si8g01880* as *SiSCR1* and *SiSCR2* at the whole-genome level [25,50,51]. The chromosomal positions of *SiSCR* genes were extracted from the foxtail millet genome annotation (.gff) in the MDSi database (http://foxtail-millet.biocloud.net/home, accessed on 12 June 2023) [25] and visualized via the Graphics module in TBtools-II (V2.310, https://github.com/CJ-Chen/TBtools-II/releases, accessed on 20 December 2022) [52]. The conserved domains of the amino acid sequence of SiSCR proteins were identified through a query using the Batch-CD-Search function of the NCBI-CDD database (https://www.ncbi.nlm.nih.gov/Structure/bwrpsb/bwrpsb.cgi, accessed on 20 December 2022) [53], and visually analyzed with TBtools (V2.310) software [52]. Initial alignment of the gene products of *SiSCR1/2*, *ZmSCR1/1h*, *OsSCR1/2*, and *AtSCR* was performed using DNAMAN 8 (Lynnon BioSoft, Gloucester, MA, USA, https://www.dnaman.net/download.html, accessed on 20 December 2022), and the results were refined in Adobe Illustrator CS6 (Adobe, https://www.adobe.com/uk/products/illustrator.html, accessed on 3 July 2025). The appropriate motifs were designated as previously described [54]. The physicochemical properties of the SiSCR peptide sequences, including the number of amino acids, molecular weight, isoelectric point, instability index, aliphatic index, and hydrophobicity index, were analyzed using ExPASy Proteomics (http://web.expasy.org/protparam/, accessed on 10 March 2023) [55]. The hydrophilicity and hydrophobicity of the SiSCR proteins were predicted and analyzed using the ExPASy Protscale (https://web.expasy.org/protscale/, accessed on 10 March 2023) [55]. The transmembrane domain of SiSCR proteins was analyzed using the TMHMM 2.0 (DTU Health Tech, https://services.healthtech.dtu.dk/services/TMHMM-2.0/, accessed on 12 March 2023). The signal peptide of SiSCR proteins was predicted using the SignalP 5.0 Server (DTU Health Tech, https://services.healthtech.dtu.dk/services/SignalP-5.0/, accessed on 12 March 2023).

### 4.2. Gene Structure and Conserved Motif Analysis of SiSCR Genes in Foxtail Millet

Gene Structure Display Server (GSDS, http://gsds.cbi.pku.edu.cn/, accessed on 23 May 2023) was used to visualize the gene structures of the *SiSCR* genes [56]. Secondary structure of SiSCR proteins was predicted using the NPSA-PRABI (http://npsa-pbil.ibcp.fr, accessed on 16 June 2024) [57]. The SiSCR protein sequences were submitted to predict the three-dimensional structure by Alphafold3 (https://alphafold.com/, accessed on 20 September 2024) [58], and the results are visualized by PyMOL (V2.5.5, Schrodinger, LLC. http://www.pymol.org/, accessed on 20 September 2024). Multiple Expectation Maximization for Motif Elucidation suite (MEME, https://meme-suite.org/, accessed on 20 May 2023) [23] was used to search for conserved motifs of SiSCR proteins. The MEME/MAST motif pattern redrawer function of TBtools (v2.310) was used to visualize the conserved motifs of SiSCR proteins [52].

### 4.3. Analysis of Promoter Cis-Regulatory Elements of SiSCR Genes in Foxtail Millet

The upstream 2500 bp sequences of the *SiSCR* transcription start site were extracted by TBtools (V2.310) and submitted to the PlantCARE database (http://bioinformatics.psb.ugent.be/webtools/plantcare/html/, accessed on 23 December 2023) [24] to predict *cis*-regulatory elements. The analysis results were plotted using TBtools (v2.310) software [52].

### 4.4. Phylogenetic Analysis of SCR in 16 Plant Species

The amino acid sequences of SCR proteins from 16 plant species were obtained using NCBI, including *Setaria italica*, *Setaria viridis*, *Panicum miliaceum*, *Zea mays*, *Sorghum bicolor*, *Oryza sativa*, *Hordeum vulgare*, *Brachypodium distachyon*, *Capsicum annuum*, *Solanum lycopersicum*, *Nicotiana tabacum*, *Arabidopsis thaliana*, *Glycine max*, *Cucumis sativus*, *Dalzellia ubonensis*, and *Gossypium hirsutum*, and used the ClustalW program to perform multiple alignments. The comparison results were analyzed using the Neighbor-Joining (NJ) method in MEGA 7 software [59], with the Bootstrap parameter 1000, to construct a phylogenetic tree.

### 4.5. Plant Materials and Treatments

For expression heatmap, transcriptome data from different tissues (roots, stems, leaves, grains, and panicles) of Jingu 21 at different developmental stages were obtained from the MDSi database (http://foxtail-millet.biocloud.net/home, accessed on 16 November 2023) [25], the accession number is CRA001954 in the Beijing Institute of Genomics Data Center (https://ngdc.cncb.ac.cn/, accessed on 16 November 2023). Germinated seeds were sampled at 3 days. Plants were sampled at the one-tip-two-leaf stage. Leaf 1 was sampled 2 days after heading. Neck panicle internodes, flag leaf, flag leaf sheath, stem, leaf 2, leaf sheath 1, and root were sampled during the filling stage. Panicle 1 are primary panicle branches, and panicle 2 are third panicle branches at the panicle–branch differentiation stage. Immature spikelets were sampled at the S2 and S4 stages. Immature seeds were sampled at the early grain filling stage (S1), middle grain filling stage (S2), late grain filling stage (S3), final grain filling stage (S4), and grain maturation stage (S5) [33]. Leaf veins are tertiary veins. The RNA-seq data of the 21 different tissues mentioned above from the MDSi database were downloaded. TPM values were calculated to assess gene expression levels, and a heatmap was generated using TBtools (v2.310) software [52].

For RT-qPCR analysis of different tissues in foxtail millet, the seedlings of Jingu 21 were cultured with Hoagland hydroponic nutrient solution (Beijing Coolaber Technology Co., Ltd., Beijing, China) and grown in an artificial climate chamber with light intensity of 30,000 LX for 16 h in the daylight at 28 °C and 8 h in the dark at 22 °C. Seeds germinated for 3 days were sampled. Roots, stems, and leaves of seedlings grown for 28 days were sampled. Immature seeds at the middle grain filling stage were sampled. Young panicles at the early growth stage were sampled. For RT-qPCR analysis of *SiSCR* genes in foxtail millet roots under different phytohormone treatments, seedlings of Yugu1 and An04 were cultured for 5 days and then transferred to Hoagland hydroponic nutrient solution containing 50 μM IAA, 25 μM GA_3_, or 100 μM MeJA. The culture conditions were consistent with those mentioned above. Seedling roots were collected at 0, 12, 24, and 48 h after the IAA, GA_3_, or MeJA treatment. The samples were frozen in liquid nitrogen and stored at -80 °C. Three biological replicates were performed for each treatment. RNA from different tissues was extracted by Trizol method using Total RNA Extract Reagent and RNA Extraction solution (Beijing Coolaber Technology Co., Ltd., Beijing, China). Reverse transcription was performed using All-in-One First-Strand Synthesis MasterMix (with dsDNase) (BestEnzymes Biotech Co., Ltd., Lianyungang, China) for Real-time Quantitative Polymerase Chain Reaction (RT-qPCR). F488 SYBR qPCR Mix (Universal) (BestEnzymes Biotech Co., Ltd., Lianyungang, China) was used as a fluorescent dye. Primers were designed using Primer 5.0 (Table S3). We used the gene *Si9g37480* as an internal control, which was stably expressed at each growth stage in almost all tissues [2]. Each reaction was performed three times, and the 2^−∆∆CT^ method [60] was used to calculate the relative gene expression levels. Statistical analysis of results was reported as means ± SE. Student’s *t*-test determines the significance.

For expression patterns, root tip samples were collected from 9-day-old Yugu1 and An04 seedlings treated with ABA (2 µM) /PEG6000 (20%) and the untreated control (CK) in an artificial climate chamber under the same conditions as mentioned above. Leaf samples were collected from 30-day-old An04 and Yugu1 plants treated with drought (D) and untreated control (CK) in the experimental field of Shanxi Agricultural University’s Minor Crops molecular Breeding Team in Taigu (112°28′ E to 113°01′ E, 37°12′ N to 37°3′ N) or in an artificial climate chamber under the same conditions as mentioned above. The 30-day-old plants were subjected to drought treatment and untreated control for 10 days, respectively, then the plants growing in the experimental field were sampled in the morning (M), at noon (N), and in the evening (E), respectively and the plants growing in the artificial climate chamber were sampled at midday (md) and in the pre-dawn (pd) period, respectively. The newest fully expanded leaves on the above-ground parts were collected. Young panicle samples were collected from Jingu21 when branch meristems were specified (stage 1, approximately 1.0–1.5 mm) and clearly formed (stage 2, approximately 2.5–3.0 mm). Subsequently, the collected roots, leaves, and young panicles were rapidly frozen in liquid nitrogen and stored at −80 °C. Novogene bioinformatics Co., Ltd. (Beijing, China) was commissioned to construct RNA libraries for high-throughput sequencing on the Illumina Hiseq platform. Total RNA was extracted from foxtail millet roots, leaves, and panicles using Trizol or magnetic bead methods. mRNA was enriched with Oligo(dT) beads, fragmented, and reverse-transcribed into cDNA. Adapters were ligated and amplified to construct libraries. Clean reads were obtained via Trimmomatic [61], aligned to the reference genome using HISAT2 (v2.0.5) [62], and TPM values were calculated with R. Genes with TPM > 10 were filtered, followed by differential expression and functional analyses. Each sample had 3 biological replicates. Statistical analysis of results was reported as means ± SE. Student’s *t*-test determines the significance.

### 4.6. Co-Expression Network Analysis of the SiSCR Genes in Foxtail Millet

To identify co-expressed gene networks associated with the target gene, we performed Weighted Gene Co-expression Network Analysis (WGCNA) using RNA-seq data from Yugu1 plants treated with or without ABA (2 µM) /PEG6000 (20%), as described in Section 4.5. The top 20 genes co-expressed with the target gene (based on weight values within the module) were selected (Table S4). These genes were then imported into Cytoscape (V3.10.2, https://cytoscape.org/, accessed on 3 July 2025) for network visualization, where they were color-coded according to their functional annotations to highlight distinct biological roles.

### 4.7. Expression Analysis of pSuper:SiSCR1/2-GFP in Arabidopsis thaliana

The *SiSCR1/2* gene open reading frame (ORF), synthesized by Sangon Biotech, was digested using the restriction enzymes *Sma*I and *Bam*HI and cloned into the *pUC57* vector to generate the recombinant plasmid *pUC57-SiSCR1/2*. Subsequently, to generate the *SiSCR1/2* overexpression construct, the *SiSCR1/2* ORF was amplified from *pUC57-SiSCR1/2* using a pair of primers (*SiSCR1/2-F/R*) and cloned into the *Hin*dIII and *Sma*I sites of the *pSuper1300-GFP* binary vector, downstream of the Super promoter. Primers are shown in Supplemental Table S3. The *SiSCR1/2-GFP* construct in *Agrobacterium tumefaciens* strain GV3101 was transformed into the *Arabidopsis* wild-type Col, and T3 homozygous transgenic positive lines were generated. Surface-sterilized T3 homozygous seeds were plated onto Murashige and Skoog (MS) medium supplemented with 2% (*w/v*) sucrose and 0.8% or 1% (*w/v*) agar. After incubation at 4 °C for 2 days, the plates were transferred to light incubators under species-specific conditions: a 16 h light/8 h dark cycle at 22 °C, 10,000 LX light, and approximately 65% relative humidity for *Arabidopsis thaliana; a* 16 h light period at 28 °C/8 h dark period at 22 °C, with 30,000 LX light, and approximately 50% relative humidity for foxtail millet (*Setaria italica*). Seedlings were grown for 5 days before phenotypic analysis. All the transgenic lines were observed and imaged using a Leica SP8 confocal microscope. GFP was excited at 488 nm, and emission was detected at 490–530 nm. Propidium iodide (Sigma-Aldrich, St. Louis, MO, USA, P4170) staining in root tips was performed as described in [63]. Propidium iodide was excited at 561 nm, and emission was detected at 575–620 nm.

### 4.8. Root Growth Analysis of Arabidopsis thaliana pSuper:SiSCR1/2-GFP Transgenic Plants

In the root growth assay, 4-day-old seedlings were carefully transferred to either ½MS medium or ½MS medium supplemented with 30 μM ABA, 1 μM IAA, 1 μM GA3, and 10 μM MeJA. The root tips were precisely aligned in a straight line. Subsequently, the culture plates were vertically positioned in a photoperiod incubator for an additional 2 days. After this incubation period, the plates were photographed, and the root growth was measured using ImageJ (V1.44p, https://imagej.net/ij, accessed on 3 July 2025) [64].

For analysis of root meristems, 4-day-old seedlings were transferred to either ½MS medium or ½MS medium supplemented with 30 μM ABA, 1 μM IAA, 1 μM GA3, and 10 μM MeJA, and were grown for another 2 days. To visualize the cells in the root tip, propidium iodide fluorescence was employed. The roots were observed and imaged using a Leica SP8 confocal microscope. The length of root meristems was measured with ImageJ (V1.44p, https://imagej.net/ij, accessed on 3 July 2025) [64]. Statistical analysis of results was reported as means ± SE. Student’s *t*-test determines the significance.

## 5. Conclusions

In this study, we conducted a comprehensive and systematic analysis of two *SiSCR* genes in foxtail millet. Amino acid sequence analysis revealed remarkable conservation at the C-terminus of SiSCR proteins when compared with ZmSCR1/1h (maize), OsSCR1/2 (rice), and AtSCR (*Arabidopsis*) proteins, and the two *SiSCR* genes showed high similarity in sequence, structure, and physicochemical properties. *Cis*-element analysis, collinearity analysis, and RT-qPCR analysis under hormone treatments suggested that *SiSCR* genes may be involved in biological processes such as hormone response, abiotic/biotic stress response, and plant growth and development. Phylogenetically, monocotyledonous and dicotyledonous *SCRs* form distinct clades, with *SiSCRs* closest to *Setaria viridis SvSCR*. ABA treatment significantly downregulated *SiSCR* gene expression in the roots of Yugu1 but not in An04. The expression patterns of *SiSCR* genes in leaves varied across sampling times (morning, noon, evening), suggesting potential circadian regulation. In *Arabidopsis*, *SiSCR* genes are expressed predominantly in root cortex/endodermal initial cells, endodermis, cortex, root cap, and stomatal complexes. Overexpression of *SiSCR* genes in *Arabidopsis thaliana* caused ABA hypersensitivity, reducing root meristem length compared to wild-type. These results provide a foundation for further in-depth exploration of the functional characterization of the *SiSCR* genes in foxtail millet.

## Figures and Tables

**Table 1:** The properties of *SiSCR* genes in foxtail millet.

Gene ID	Gene Name	Chromosomal Location	CDS Length	Molecular Weight (KD)	Isoelectric Point	Instability Index	Aliphatic Index	GRAVY
Si7g30800	*SiSCR1*	7: 34622075-4625670 (+)	2016 bp	71.78	6.06	56.39	86.26	−0.193
Si8g01880	*SiSCR2*	8: 758375-761643 (−)	2001 bp	71.12	5.97	57.40	87.48	−0.181

**Table 2:** The secondary structure prediction of SiSCR proteins in foxtail millet.

Protein	Alpha Helix (%)	Beta Sheet (%)	Extended Strand (%)	Random Coil (%)
SiSCR1	47.30	3.60	9.31	39.79
SiSCR2	46.05	3.58	9.24	41.13

## Data Availability

The data are contained within the article and the Supplementary Materials.

## Supplementary Materials

The following supporting information can be downloaded at: https://www.mdpi.com/article/10.3390/plants14142151/s1, Figure S1: Chromosomal localization and conserved domain analysis of *SiSCR* genes; Figure S2: Sequence analysis of SiSCR proteins in foxtail millet; Figure S3: Analysis of *SiSCR* genes structures and conserved motifs; Figure S4: Conserved motif sequences analysis of SiSCR proteins in foxtail millet; Figure S5: The relative expression level of the *SiSCR* genes detected by qPCR; Figure S6: The relative expression level of the *SiSCR* genes, and detected by RT-qPCR, under treatments with IAA, GA_3_, and MeJA phytohormones; Figure S7: Expression patterns of *SiSCR* genes in young panicles of foxtail millet; Figure S8: Response of roots and root tip meristematic zones to IAA, GA_3_, and MeJA in *Arabidopsis thaliana* plants overexpressing the *SiSCR* genes. Table S1: *SCR* genes used for phylogenetic tree in 16 plant species; Table S2: The Transcripts Per Million (TPM) values of the *SiSCR* genes in different tissues; Table S3: Primer sequences used in the paper; Table S4: Co-expression networks of *SiSCR1* and *SiSCR2* under ABA and PEG6000-induced drought stress in foxtail millet.
