# Supplementary material for: Tissue-Specific Expression Analysis and Functional Validation of SiSCR Genes in Foxtail Millet (Setaria italica) Under Hormone and Drought Stresses, and Heterologous Expression in Arabidopsis
Source: Plants (Basel). 2025 Jul 11;14(14):2151. doi: 10.3390/plants14142151 (PMC12300342; doi:10.3390/plants14142151)
Supplement: Supplementary file 1 [file plants-14-02151-s001.zip › Supplementary figures .pdf]

Supplementary materials:

Figure：

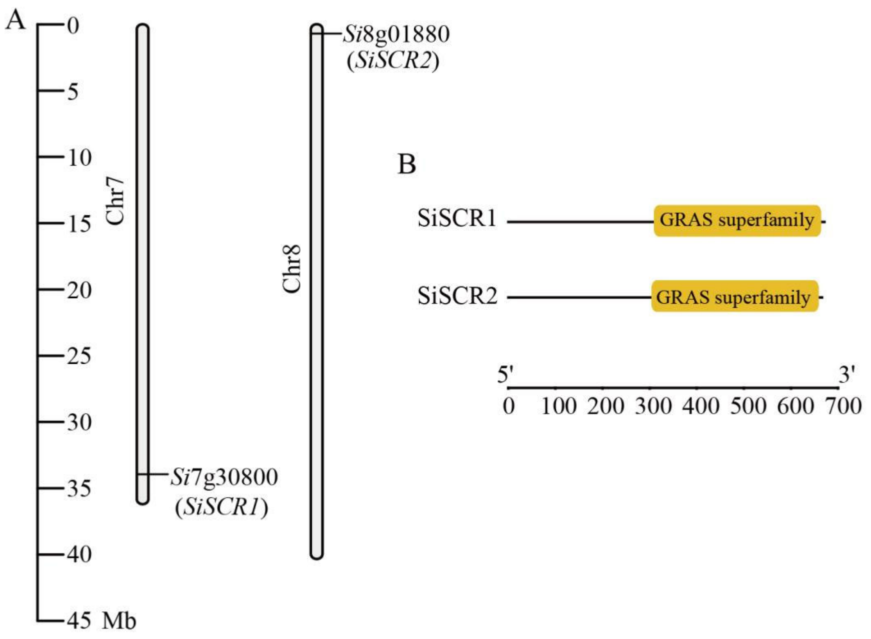

Figure S1. Chromosomal localization and conserved domain analysis of *SiSCR* genes. (**A**) Chromosomal localization. (**B**) Conserved domain analysis**.**

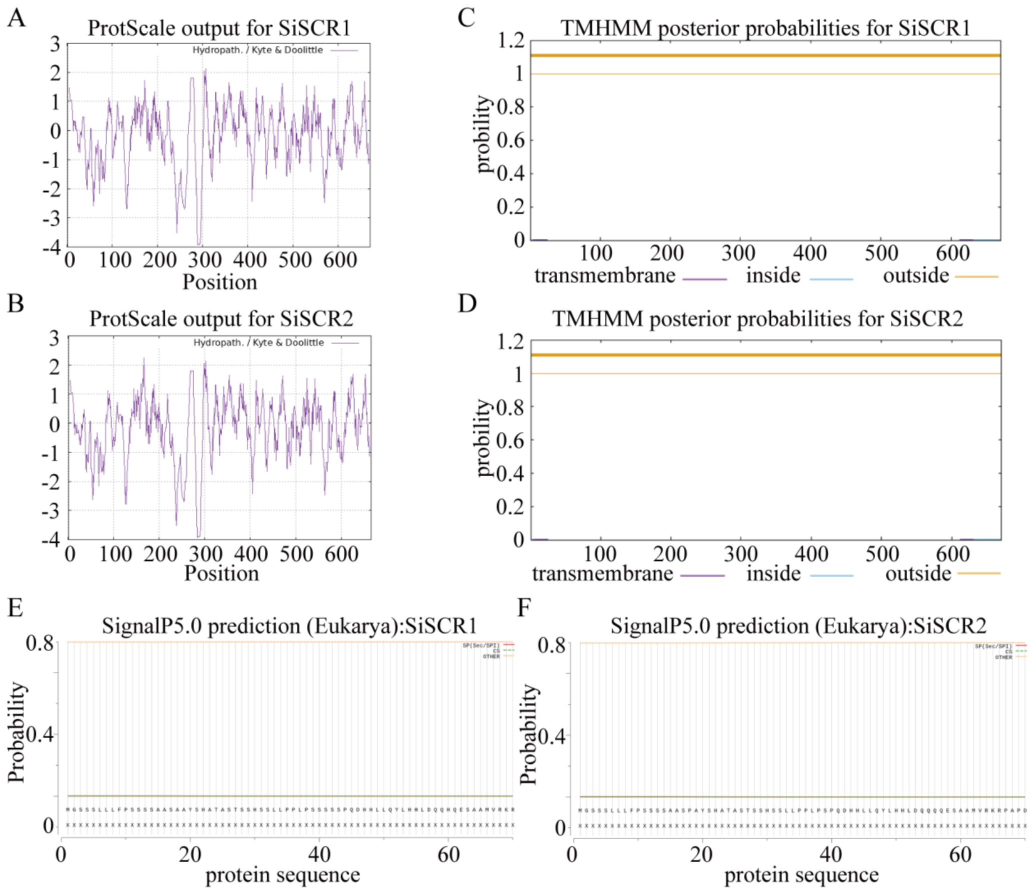

Figure S2. Sequence analysis of SiSCRs in foxtail millet.

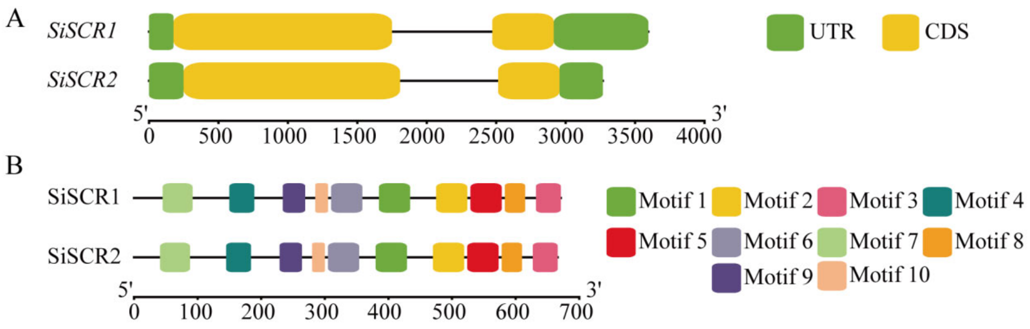

Figure S3. Analysis of *SiSCR* genes structures and conserved motifs. (**A**) Gene structure. Coding sequences (CDS) and untranslated regions (UTRs) are represented by different colored boxes, and introns are indicated by lines. (**B**) Conserved motifs. Conserved motifs within the *SiSCR* genes are represented by different colored boxes. The weblogo plots of the ten conserved motifs are shown in Figure S4.

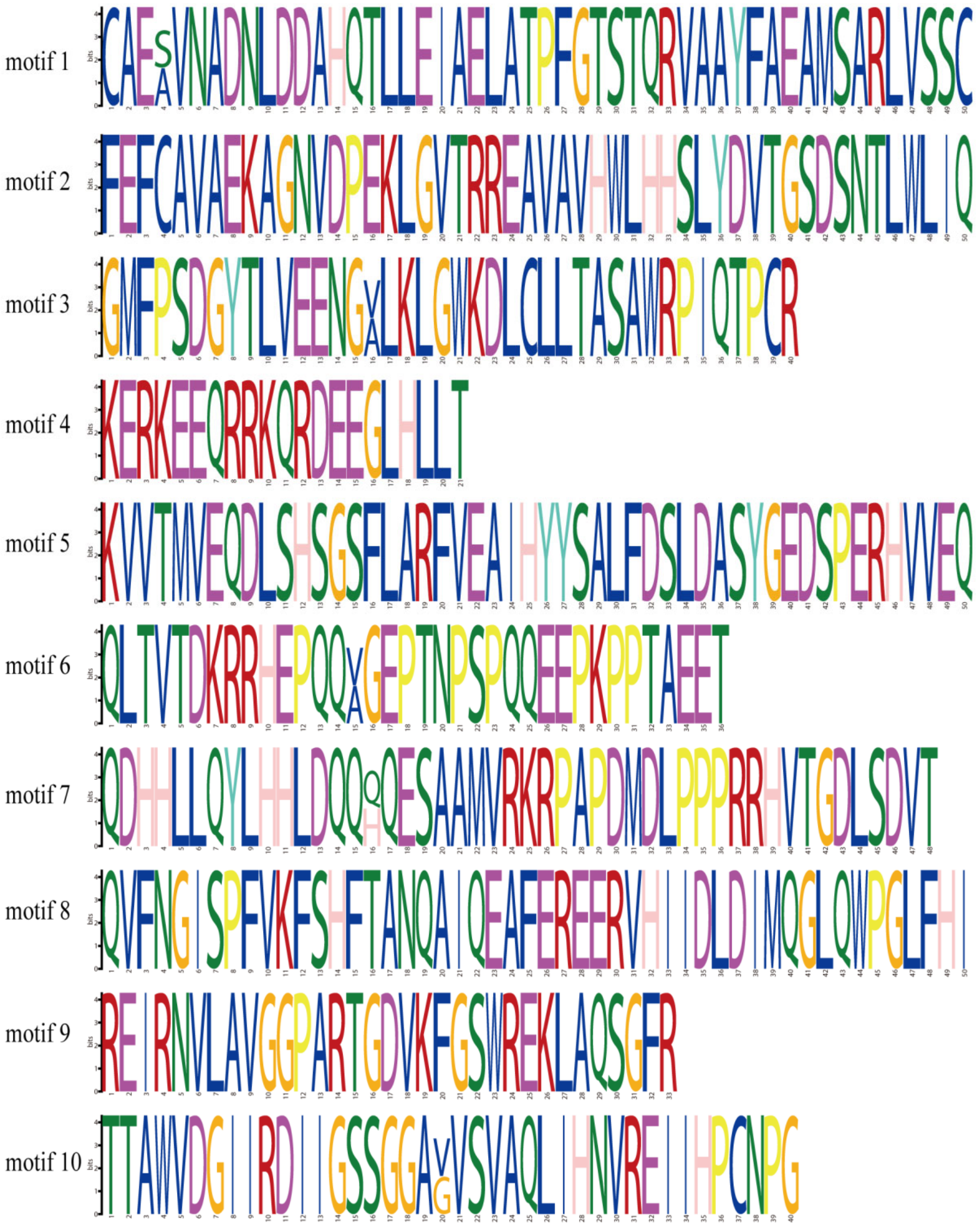

Figure S4. Analysis of conserved motif sequences for SiSCR proteins in foxtail millet

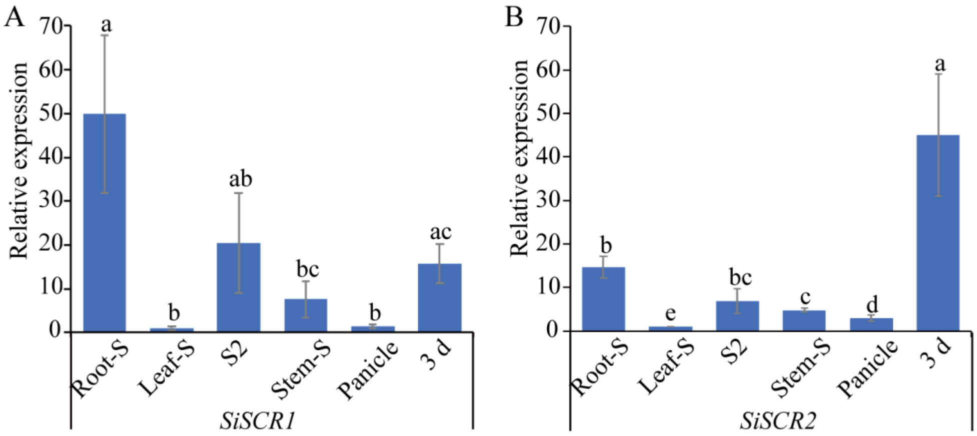

Figure S5. The relative expression level of the *SiSCR* genes detected by qPCR. Root-S, leaf-S, and stem-S were sampled from seedlings grown for 28 days. 3 d: seeds germinated for 3 days. S2: immature seeds at the middle grain filling stage. Panicle: young panicles at the early growth stage. Statistical significance was determined by *t*-test (lowercase letters indicate $p < 0.05$).

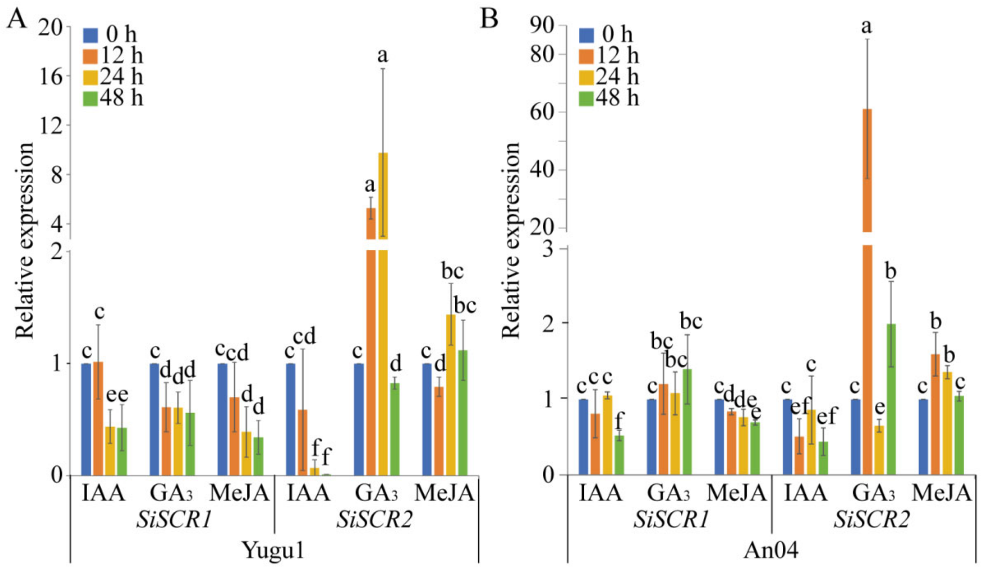

Figure S6. The relative expression level of the *SiSCR* genes, and detected by RT-qPCR, under treatments with IAA, GA_3, and MeJA phytohormones. The unstressed level (0 h) was used as a control. Statistical significance was determined by *t*-test (lowercase letters indicate $p < 0.05$).

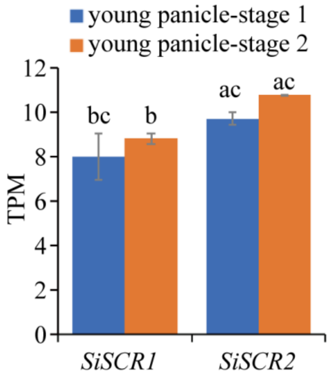

Figure S7. Expression patterns of *SiSCR* genes in the young panicles. Statistical significance was determined by *t*-test (lowercase letters indicate $p < 0.05$). TPM: transcripts per million.

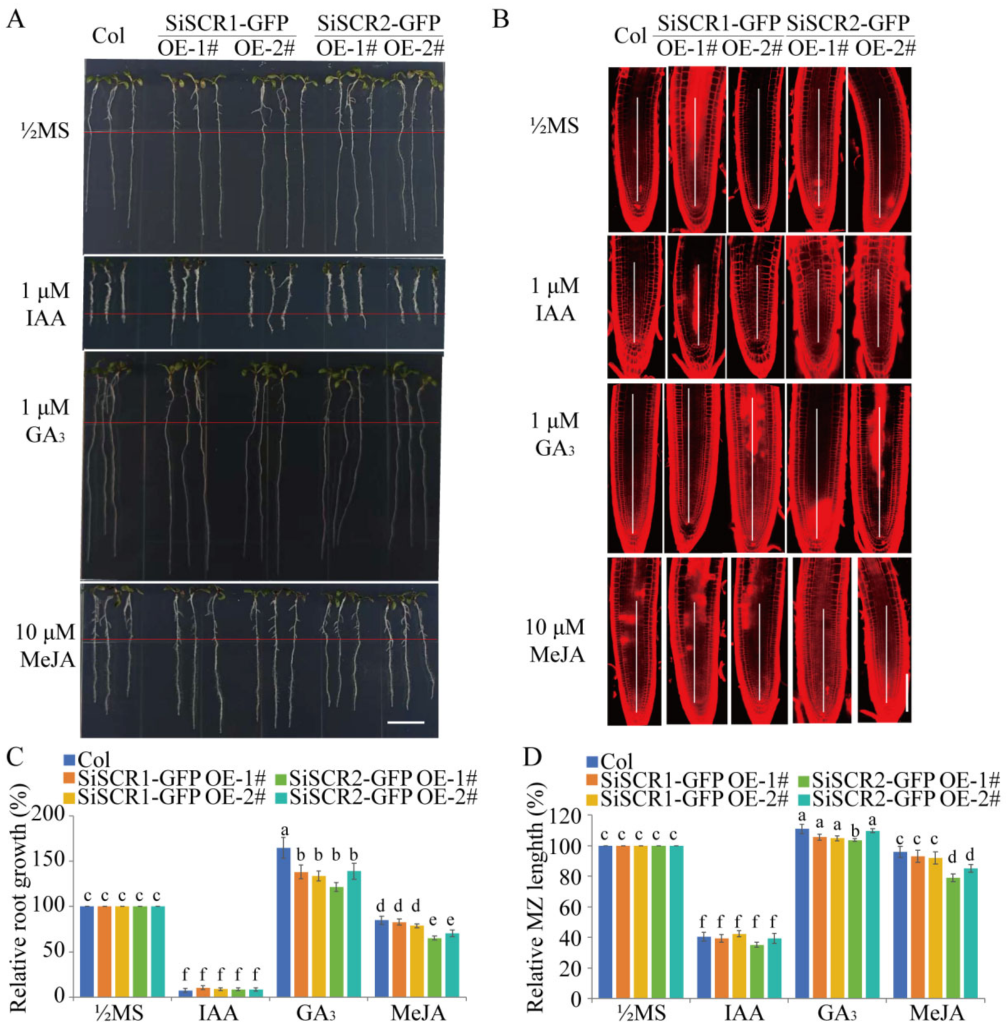

Figure S8. Response of roots and root tip meristem zones to IAA, GA_3, and MeJA in *Arabidopsis thaliana* plants overexpressing the *SiSCR* genes. (A) Primary root phenotypes of wild-type Col, *SiSCR1*-overexpressing lines (OE-1# and OE-2#) and *SiSCR2*-overexpressing lines (OE-1# and OE-2#). Seeds were germinated on ½MS medium for 4 days and then transferred to fresh ½MS medium and ½ MS medium with IAA, GA_3 or MeJA for 2 days (Bar=1 cm). (B) Relative primary root growth of plants in (A), normalized to root length on ½MS medium (set as 100%). Data represent mean ± SE (n=3 replicates; 5 seedlings per replicate). (C) The root meristematic zones of wild-type Col, *SiSCR1*-overexpressing lines (OE-1# and OE-2#), and *SiSCR2*-overexpressing lines (OE-1# and OE-2#) on the ½MS medium and the ½MS medium with IAA, GA_3 or MeJA (Bar = 100 μm). (D) Relative meristematic zone (MZ) length of plants in (C). All experiments were repeated three times with consistent results. Statistical significance was determined by *t*-test (lowercase letters indicate $p < 0.05$).
